# Population Genomic Insights into Genetic Differentiation and Environmental Adaptation of the Asian Honey Bee (*Apis cerana*) in Sichuan, China

**DOI:** 10.3390/insects17101022

**Published:** 2026-10-03

**Authors:** Xiangcheng Xu, Dingyuan Ma, Bingrong Liu, Mingxian Yang, Kang Lai, Jiandong Yang

**Affiliations:** 1 College of Animal Science and Technology, Sichuan Agricultural University, Chengdu 611130, China; yu292776@gmail.com (X.X.); qqqqqmartin@163.com (D.M.);; 2 Sichuan Provincial Animal Husbandry Master Station, Chengdu 610041, China

**Keywords:** *Apis cerana*, population genomics, genetic differentiation, environmental adaptation

## Abstract

The Asian honey bee (*Apis cerana*) is a key native pollinator in Asia, contributing substantially to both natural and agricultural ecosystems. However, climate change and human activities increasingly threaten its pollination services and long-term survival. Understanding patterns of genetic differentiation and local adaptation is therefore essential for protecting native populations. Sichuan Province in China, with its complex mountains, deep valleys, and pronounced climatic heterogeneity, offers an excellent natural laboratory for studying these processes in *A. cerana*. We collected 139 worker bees from 22 locations across Sichuan and generated whole-genome resequencing data. By integrating these data with genomes from eight well-established subspecies, we show that bees from Xinlong, Jiuzhaigou, and Kangding form three genetically differentiated populations that are distinct from all known subspecies. These populations display moderate to high genetic differentiation and unique demographic histories relative to previously described subspecies. Selective-sweep analyses further identified 200–300 candidate genes under positive selection in each population. These genes are enriched in functions related to chemical sensing, flight musculature, cell-cycle regulation, osmotic balance, and stress response, consistent with adaptation to patchy nectar resources, a short spring reproductive window, and large diurnal temperature swings. Our findings uncover three previously unrecognized genetic resources of *A. cerana* and provide genomic insights into their local adaptation, offering a valuable foundation for conservation and breeding programs.

## Simple Summary

Sichuan Province, China, is characterized by complex topography and diverse climatic conditions that create a mosaic of habitats for the Asian honey bee (*Apis cerana*). These environments may have fostered distinct genetic groups that have differentiated and adapted to local conditions. Understanding such genetic differentiation and environmental adaptation is essential for maintaining the genetic integrity of native populations and designing effective conservation strategies. In this study, we collected 139 *A. cerana* samples from 22 locations across Sichuan Province and integrated their whole-genome resequencing data with those of eight well-established *A. cerana* subspecies. We found that bees from Xinlong, Jiuzhaigou, and Kangding form three genetically differentiated populations that are distinct from known subspecies. These populations also exhibit moderate to high genetic differentiation and unique demographic histories. Selective-sweep analyses identified hundreds of genes under positive selection in each population; these genes are likely associated with adaptation to patchy nectar resources, a short spring reproductive window, and large diurnal temperature fluctuations. Our findings provide important references for the conservation of *A. cerana* and the future utilization of its genetic resources.

## 1. Introduction

The Asian honey bee (*Apis cerana*) is an important native pollinator throughout Asia. Relative to the western honey bee (*Apis mellifera*), it possesses several advantageous traits, including the ability to exploit scattered nectar and pollen resources, resistance to mites and diseases, strong stress tolerance, low feed consumption, a long foraging season, and broad environmental adaptability [1]. It has long provided essential pollination services in both natural and agricultural ecosystems. However, climate change, pesticide use, and habitat loss and fragmentation now pose significant threats to the survival of *A. cerana* populations [2,3,4]. In addition, the expansion of commercial beekeeping has led to the introduction of non-native colonies and frequent long-distance transport, which can cause genetic admixture and erode the integrity of local *A. cerana* genetic resources [5,6]. Consequently, clarifying patterns of population genetic differentiation and the genomic basis of environmental adaptation in *A. cerana* is critical for identifying distinct genetic units, preserving native genetic integrity, and formulating targeted conservation strategies.

Previously, the two most systematic studies of population differentiation in the Asian honey bee—one based on mitochondrial DNA (mtDNA) and the other on morphometrics—identified five and six major groups, respectively, across the species’ range [7,8,9]. In recent years, high-throughput sequencing, particularly whole-genome resequencing, has greatly enhanced our ability to characterize genetic variation at the genome-wide scale [10,11]. Analyzing 343 genomes of the widely distributed Asian honey bee, Ji et al. [12] proposed that multiple peripheral groups radiated from a central ancestral population and independently adapted to diverse habitats. Building on these findings, Qiu et al. [13] expanded the geographic sampling and formally defined eight subspecies of mainland Asian honey bees. However, this eight-subspecies framework was established primarily through representative sampling at broad geographic scales and therefore mainly captures continental-scale patterns of differentiation. Additional analyses are needed to resolve regional-scale structure shaped by topographic barriers and environmental heterogeneity. Physical barriers such as mountains and straits have been shown to restrict gene flow and drive genetic differentiation in *A. cerana*. For example, whole-genome resequencing of 180 individuals by Chen et al. [14] demonstrated that population differentiation is driven mainly by physical barriers rather than geographic distance alone. Consequently, genetic differentiation and adaptive divergence of Asian honey bees deserve particular attention in regions that combine pronounced geographic barriers with complex environmental gradients.

Sichuan lies in the transition zone between the first and second steps of China’s terrain and exhibits a pronounced west-high, east-low topographic pattern. It comprises three major natural units: the Sichuan Basin, the northwestern Sichuan Plateau, and the mountains of southwestern Sichuan. The southwestern portion belongs to the northern section of the Hengduan Mountains, where high mountains, deeply incised valleys, and longitudinal river systems create distinct vertical climatic zones and habitat differentiation [15,16]. This complex landform system of plateaus, mountains, deep valleys, basins, and hills provides natural conditions that both restrict gene flow and impose heterogeneous environmental selection on Asian honey bee populations [17,18]. Studies of gorge landscapes on the southeastern margin of the Tibetan Plateau have shown that even *A. cerana* populations from adjacent valleys can exhibit significant genetic differentiation owing to restricted gene flow [19]. These observations suggest that Sichuan’s complex topography may harbor distinct genetic groups that have differentiated and adapted to different habitats.

To date, studies of *A. cerana* genetic resources in Sichuan have relied mainly on morphology, mtDNA, and microsatellite markers [20,21,22]. Limited by the resolution of these approaches and the amount of genetic information they capture, existing work remains insufficient for resolving fine-scale population genetic structure or the genomic basis of adaptation to complex environments. Consequently, broad-scale sampling combined with whole-genome resequencing data is still needed to clarify patterns of genetic differentiation among *A. cerana* populations from different geographic units within Sichuan Province and to determine whether under-recognized genetic resources exist in the region.

Based on this background, the present study focused on *A. cerana* populations in Sichuan. Using extensive sampling and whole-genome resequencing, we systematically characterized population genetic structure and differentiation, compared the Sichuan samples with known *A. cerana* subspecies, and tested whether the province harbors previously under-recognized genetic resources. We further performed selective-sweep and functional-enrichment analyses on the newly identified genetic populations to identify candidate genes associated with environmental adaptation. The results provide an important foundation for the identification, conservation, and future utilization of *A. cerana* genetic resources in Sichuan, as well as for a broader understanding of genetic differentiation in the species worldwide.

## 2. Materials and Methods

### 2.1. Sample Collection

**Figure 1:**
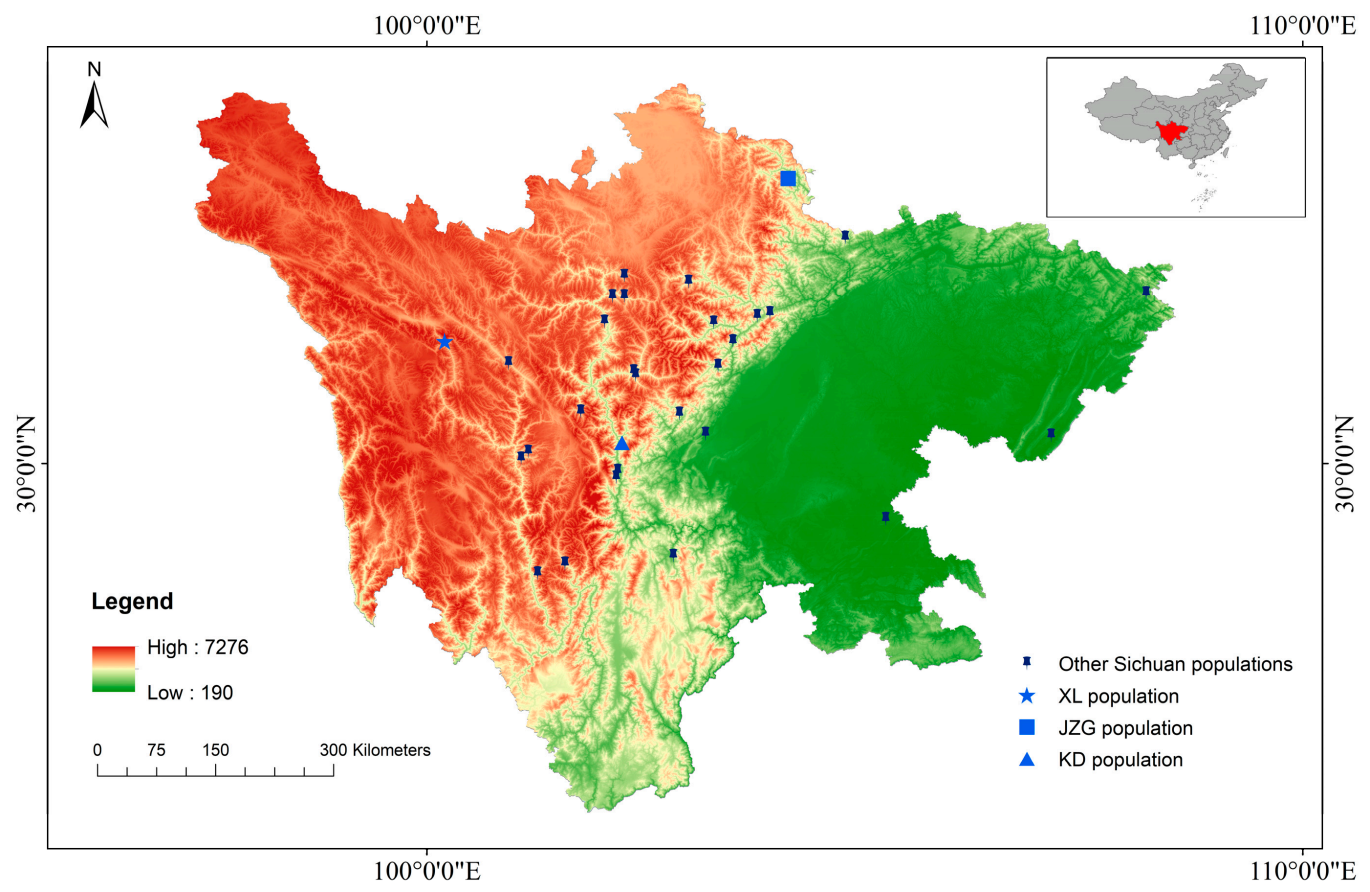
Geographic distribution of *A. cerana* sampling sites in Sichuan Province, China. The background color gradient indicates elevation, and the inset shows the location of Sichuan Province within China. Symbols denote the sampled populations: star, Xinlong (XL); square, Jiuzhaigou (JZG); triangle, Kangding (KD); pin, other Sichuan populations.

A total of 139 *A. cerana* worker bee samples were collected from 22 representative geographic populations in Sichuan Province, China, covering multiple ecological gradients and landform units (Table S1 and Figure 1). The sampling sites included the Qionglai Mountains region (Maerkang, Jinchuan, Xiaojin, Heishui, Li County, and Baoxing), the Dadu River Basin (Danba, Luding, and Ganluo), the Daxue Mountains region, including Daofu, Kangding (KD), and Jiulong, and the Yalong River Basin, including Xinlong (XL) and Yajiang. They also covered the Minjiang River Basin (Qionglai, Wenchuan, and Mao County), the margin of the Sichuan Basin (Fushun), the Minshan Mountains region (Qingchuan), the Minshan Mountains–upper Jialing River region, including Jiuzhaigou (JZG), the watershed region between the Hanjiang and Jialing rivers (Wanyuan), and the Huaying Mountains–Qujiang River Basin (Linshui). To improve sampling quality and representativeness, wild colonies in remote mountainous areas were selected. At least five colonies were selected in each collection site and one worker bee from each colony was used for whole-genome resequencing. During sampling, GPS was used for precise positioning, and environmental information, including altitude, habitat type, and vegetation conditions, was systematically recorded. After collection, all samples were immediately immersed in absolute ethanol, placed in sealed glass bottles with ground-glass stoppers, brought back, and stored at −20 °C.

### 2.2. DNA Extraction and Whole-Genome Resequencing

DNA was extracted from thoracic tissue using a magnetic bead-based genomic DNA extraction kit (Tiangen Biotech, Beijing, China; DP705) according to the manufacturer’s instructions. The quality of the extracted total DNA was assessed using agarose gel electrophoresis, NanoDrop (Thermo Fisher Scientific, Wilmington, DE, USA), and Qubit 2.0, and samples meeting the library construction requirements were used for subsequent analysis. For library construction, DNA fragments generated by Covaris ultrasonic shearing (Covaris, Woburn, MA, USA) were subjected to end repair, A-tailing, adapter ligation, purification, and PCR amplification. Library concentration and insert size were then assessed using Qubit and Agilent 2100 (Agilent Technologies, Waldbronn, Germany), and accurate quantification was performed by qPCR. Qualified libraries were pooled according to the target sequencing data output and subjected to paired-end sequencing on the Illumina HiSeq platform (PE150; completed by Novogene, Beijing, China).

### 2.3. Public Data

To compare the genetic resources of *A. cerana* in Sichuan with the eight known subspecies, we selected 40 samples from the publicly available whole-genome resequencing data released by Ji et al. [12], including five samples from each subspecies (Table S2). These samples covered the major subspecies: Hainan (*Apis cerana hainana*, HN), Northeast China (*Apis cerana japonica*, NE), Qinghai (*Apis cerana guidensis*, QH), Aba (*Apis cerana abansis*, AB), Taiwan (*Apis cerana taiwanensis*, TW), Bomi (*Apis cerana skorikovi*, BM), Central China (*Apis cerana cerana*, CT) from China, and Malaysia (*Apis cerana cerana*, ML). The raw sequencing data were downloaded from the NCBI Sequence Read Archive (SRA) under project accession number PRJNA592293 (Table S2). All public data were reprocessed using the same quality control, alignment, variant calling, and filtering pipeline as the newly sequenced samples in this study to ensure comparability in subsequent joint analyses.

### 2.4. Data Preprocessing and Quality Control

To improve the reliability and accuracy of the raw high-throughput sequencing data, systematic data preprocessing was performed for all paired-end sequencing samples, with a focus on reducing potential interference from adapter contamination, low-quality bases, and short reads during subsequent alignment and variant calling. Preprocessing was performed in paired-end (PE) mode using Trimmomatic software (version 0.39) [23], with the TruSeq2-PE adapter database used for adapter identification and removal. The core parameters included LEADING:3, which removes bases with a quality score below Q3 at the beginning of reads, and MINLEN:70, which filters out reads shorter than 70 bp. During preprocessing, both paired reads and unpaired surviving reads were retained to maximize the preservation of valid data.

### 2.5. Genome Alignment and BAM File Standardization

After quality control of the sequencing data, the cleaned reads were aligned to the reference genome using the BWA-MEM algorithm (version 0.7.12) [24]. The reference genome used in this study was GCA_029531695.1, with a genome size of approximately 217.7 Mb [25]. During alignment, complete Read Group (RG) information was embedded in the format @RG\tID:{sample ID}\tLB:{library ID}\tSM:{sample name}\tPL:ILLUMINA to ensure traceability of sample origin and compatibility with the metadata structure required by downstream analysis tools such as GATK. The SAM files generated from alignment were converted to BAM format using SAMtools (version 1.7) [26], and sorting (SortSam) and duplicate marking (MarkDuplicates) were performed using the Picard toolkit (version 2.25.7). The identification and marking of duplicate reads were mainly used to reduce systematic bias introduced during PCR amplification and improve the accuracy of subsequent variant calling. The MarkDuplicates output included the deduplicated BAM file (sorted_dedup.bam), index file (.bai), and duplicate-marking metrics file (.metrics), which were used for quality assessment. In addition, to ensure the integrity and compatibility of the reference genome, a FASTA index was generated using samtools faidx (SAMtools version 1.7), and a .dict file was generated using the Picard CreateSequenceDictionary (Picard version 2.25.7) module, ensuring that the reference sequences had a GATK-compatible indexing system.

### 2.6. Single-Sample Variant Calling and Joint Genotyping

Variant calling was performed on the sorted and duplicate-marked BAM files using the HaplotypeCaller module in GATK software (version 4.1.4.0) [27]. To support subsequent multi-sample joint analysis, variant calling was run in GVCF mode (-ERC GVCF), generating a GVCF file (.g.vcf.gz) and the corresponding index file for each sample. After sample-level variant calling was completed, all single-sample GVCF files were combined using the GATK CombineGVCFs module, followed by joint genotyping with the GenotypeGVCFs module to generate a standardized VCF file (genome.vcf) containing single nucleotide polymorphism (SNP) and insertion/deletion (INDEL) information for all samples. The GATK SelectVariants tool was used to separate variant types from the joint genotyping results, and SNP and INDEL subsets were extracted separately for subsequent analyses.

### 2.7. SNP Selection and Filtering

Multiple quality control thresholds were applied to filter the SNP dataset using the VariantFiltration module in GATK (version 4.1.4.0). The filtering criteria were as follows: variant confidence normalized by depth (QD) was required to be no lower than 2.0; mapping quality (MQ) was required to be no lower than 40.0; the Phred-scaled strand bias value (FS) was required to be no higher than 60.0; the Strand Odds Ratio (SOR) was required to be lower than 3.0; the mapping quality rank sum test value for the difference between alternative and reference alleles (MQRankSum) was required to be greater than −12.5; and the read position rank sum test value (ReadPosRankSum) was required to be greater than −8.0. These metrics are widely accepted standards in current population variant studies. In addition, to further improve the population representativeness and analytical stability of variant sites, the filtered VCF file was subjected to secondary cleaning using VCFtools (version 0.1.16) [28]. The parameters included a minor allele frequency (MAF) threshold of no lower than 0.05 to remove low-frequency noisy variants; a site missing rate of no higher than 20% (--max-missing 0.8) to ensure that each site covered a sufficient number of samples; a sequencing depth range of 2–1000; with the minimum depth threshold used to exclude genotypes with insufficient read support and the maximum depth threshold of 1000 used to exclude extremely high-coverage genotypes potentially associated with repetitive sequences, paralogous regions, or nonspecific read mapping; a variant quality (QUAL) threshold of no lower than 30; no additional stringent genotype quality threshold was imposed (minGQ = 0), because the SNP dataset had already undergone GATK VariantFiltration and was further controlled by QUAL, sequencing depth, site missing rate, MAF, and biallelic SNP filtering, thereby avoiding excessive loss of genotype information caused by further increasing the GQ threshold; and retention of only biallelic SNPs to avoid the influence of complex allelic patterns on subsequent analyses.

### 2.8. Principal Component Analysis (PCA)

To reveal the overall pattern of genetic differentiation and potential clustering structure among samples, principal component analysis (PCA) was performed on the high-quality SNP dataset. Prior to PCA, the high-quality SNP dataset was subjected to linkage disequilibrium (LD) pruning using PLINK with the parameter --indep-pairwise 50 10 0.4, corresponding to a 50-SNP sliding window, a step size of 10 SNPs, and an *r*^2^ threshold of 0.4. The resulting LD-pruned SNP dataset was used for PCA. The analysis was conducted using PLINK (version 1.9), allowing nonstandard chromosome names (--allow-extra-chr) and treating half-called genotypes as missing (--vcf-half-call missing). To ensure standardized site information, variants without IDs were uniformly named using --set-missing-var-ids @:#. The first 10 principal components were extracted as representative variables for population genetic structure analysis, and the corresponding eigenvectors and eigenvalues were output as plink_pca.eigenvec and plink_pca.eigenval files, respectively. The eigenvectors were used to visualize genetic distances among samples, whereas the eigenvalues were used to evaluate the explanatory power of each principal component and assist in assessing the rationality of the number of principal components retained. In subsequent graphical visualization, the eigenvectors were combined with sample grouping information and presented as scatter plots, providing a structural basis for subsequent population assignment and clustering interpretation.

### 2.9. Ancestry Component Analysis

To further resolve the admixed genetic composition of the samples and identify potential ancestral populations, maximum likelihood estimation was performed on the SNP data using ADMIXTURE software (version 1.3.0) [29]. To reduce the influence of linkage disequilibrium (LD) on the accuracy of model inference, the SNP dataset was subjected to LD pruning using PLINK before analysis. Specifically, a sliding-window method (--indep-pairwise 50 10 0.4) was applied, in which *r*^2^ was evaluated every 10 SNPs within a window of 50 SNPs, and sites with *r*^2^ less than 0.4 were retained as independent SNPs for model training. After LD filtering, the SNP data were converted into PLINK binary format (BED) and input into ADMIXTURE to estimate multiple candidate numbers of ancestral populations (K values) and perform cross-validation analysis. Values of K ranging from 2 to 15 were tested, and five independent runs were performed for each K value. A 10-fold cross-validation procedure was applied in each run. The optimal K value was determined primarily based on the cross-validation (CV) error, with the K value showing the lowest CV error considered optimal. The QC.{K}.Q files were merged with the sample information file (QC.fam) for downstream visualization and comparison.

### 2.10. Phylogenetic Tree Construction and Visualization

A maximum likelihood (ML) phylogenetic tree was constructed based on the high-quality SNP data. First, the standard VCF file was converted into PHYLIP format to meet the input requirements for phylogenetic analysis. After conversion, maximum likelihood tree inference was performed under a DNA model using IQ-TREE 2 software (version 2.2.0) [30]. The model was set to GTR (General Time Reversible), and 1000 ultrafast bootstrap replicates (-B 1000) were performed with the branch optimization option enabled (-bnni) to improve branch support and tree robustness. During the analysis, 50 threads and a maximum memory limit of 8G (-mem 8G) were used for efficient computation. The resulting tree file was output in standard Newick format and uploaded to the Interactive Tree of Life (iTOL v7; https://itol.embl.de/, accessed on 20 September 2025) platform for visual rendering. Population annotation information and ancestry component data were combined to provide an integrated display of evolutionary relationships and population differentiation patterns.

### 2.11. Population Genetic Polymorphism and Differentiation Indices

The genetic differentiation index (*F*_ST_) among populations was calculated using the Weir and Cockerham method [31] to evaluate the level of genomic differentiation between populations. Whole-genome SNP data were obtained from the VCF file after strict quality filtering. Individuals were assigned to different populations according to sample origin information, and sample lists were generated. Genome-wide *F*_ST_ was calculated using a sliding-window approach, and Weir and Cockerham *F*_ST_ estimation was implemented using VCFtools (version 0.1.17) [28]. The window size was set to 50 kb, with a step size of 10 kb, allowing continuous scanning across the genome to smooth local signals. During the analysis, only biallelic SNP sites that passed quality control were retained to identify genomic regions with high levels of genetic differentiation. The resulting windowed *F*_ST_ values were used to evaluate genomic differentiation patterns among populations and to screen candidate regions potentially affected by differential selection.

### 2.12. Linkage Disequilibrium (LD) Decay Analysis

To investigate linkage disequilibrium (LD) patterns in *A. cerana* populations from different regions of Sichuan, LD decay analysis was performed for each population to evaluate recombination patterns and genetic diversity characteristics. The analysis was conducted using quality-controlled SNP data in VCF format, and individuals were assigned to different populations according to sample grouping information. First, bcftools (version 1.21) [32] was used to extract sample data corresponding to each population from the whole-genome VCF file, generating population-specific VCF files. Subsequently, tabix (version 1.21) [33] was used to index the VCF files to improve data reading efficiency. LD decay was calculated using PopLDdecay v3.43 [34], with the maximum distance between SNPs set to 300 kb. Within this range, linkage disequilibrium between paired SNPs was calculated as *r*^2^ values, and the decay trend with increasing physical distance was summarized. Finally, the Perl script Plot_MultiPop.pl in PopLDdecay was used to integrate and visualize the LD decay results of different populations, generating comparative curves of *r*^2^ changes with physical distance.

### 2.13. Population History Simulation Analysis

To reconstruct historical changes in effective population size (*N*_e_), the Pairwise Sequentially Markovian Coalescent (PSMC) method [35] was used to infer historical changes in *N*_e_. This method reconstructs historical population dynamics based on information from the distribution of heterozygous sites along individual genomes. Individuals were assigned to different populations according to sample origin information, and sample lists were generated for each population. A consensus diploid sequence was constructed separately for each individual, and PSMC v0.6.5-r67 was run independently to obtain the corresponding .psmc output files, which represented individual-level trajectories of historical population size changes. To assess the uncertainty and stability of the inferred demographic trajectories, the .psmcfa sequences were first divided into segments using the splitfa utility included in PSMC v0.6.5-r67 to obtain sequence fragments for bootstrap resampling. Subsequently, 100 bootstrap replicates were performed independently for each individual using the PSMC bootstrap mode with the parameters -b -N30 -t7 -r2 -p “4 + 25*2 + 4 + 6”. To obtain population-level historical dynamics, the .psmc results of all individuals within the same population were combined and used to plot population-level effective population size change curves. The results were visualized using the psmc_plot.pl script, with the mutation rate set to μ = 3.6 × 10^−9^/site/generation based on the directly estimated mutation rate reported for bumblebees [36] and the generation time set to g = 1 year following a previous PSMC study of *A. cerana* [37], thereby converting the time scale and population size estimates into actual biological units.

### 2.14. Selective Sweep Analysis

To identify candidate selective sweep regions in local genetic populations from Sichuan, the Central population was selected as the reference population based on the results of population structure analysis. Selective sweep scans were then performed separately for the three genetic populations identified in Sichuan, namely Xinlong, Jiuzhaigou, and Kangding. First, the genetic differentiation index (*F*_ST_) and nucleotide diversity ratio (π_CT_/π_target_) were calculated between the Central population and each of the Xinlong, Jiuzhaigou, and Kangding populations, where π_target_ represents the nucleotide diversity of the corresponding Sichuan population. The overlapping regions among the top 5% windows with high *F*_ST_ and high π_CT_/π_target_ values were taken as preliminary candidate selection regions. The top 5% threshold was used as an empirical cutoff to identify genomic regions exhibiting extreme genetic differentiation or selection signals relative to the genome-wide background distribution [38,39]. Subsequently, SweeD software (version 4.0.0) [40] was used to perform composite likelihood ratio (CLR) scans separately for the Xinlong, Jiuzhaigou, and Kangding populations, and regions with CLR values in the top 5% were defined as within-population candidate selection regions. Finally, the intersection between candidate regions obtained from inter-population comparisons and those obtained from within-population CLR scans was defined as the final set of candidate selective sweep regions. This multi-statistic screening strategy was used to reduce potential false positives associated with reliance on a single statistic and to improve the robustness and reliability of candidate selective sweep identification. Demographic history and population structure may also contribute to genomic patterns interpreted as signatures of selection. Accordingly, the overlapping regions supported by multiple methods were treated as candidate selective sweep regions with relatively strong evidence of selection. Genes located within these regions were extracted according to the reference genome annotation information, defined as candidate genes under selection, and used as the target gene set for enrichment analysis. The background gene set was defined as all annotated genes in the *A. cerana* genome. Functional annotation of the background gene set was obtained using eggNOG-mapper [41], which provides comprehensive homology searches as well as Gene Ontology (GO) and Kyoto Encyclopedia of Genes and Genomes (KEGG) annotation information. To standardize the annotation format, the eggNOG-mapper auxiliary function under the “Common Tools” module in TBtools (version 0.665) [42] was used to standardize gene IDs and GO and KEGG terms, thereby ensuring consistency of annotation information across the dataset.

## 3. Results

### 3.1. Whole-Genome Resequencing of A. cerana Samples from Sichuan

Whole-genome resequencing was performed on 139 *A. cerana* samples collected from 22 locations in Sichuan Province, generating a total of 658.7 GB of raw data, with an average sequencing depth of 20× (Table S1). In addition, whole-genome resequencing datasets for 40 *A. cerana* samples from previous studies, representing eight well-established subspecies with five individuals per subspecies, were downloaded from a publicly available database. Overall, 179 individuals were included in this study, with geographic distributions covering mainland China, Hainan, Taiwan, Malaysia, and other regions (Tables S1 and S2). The average sequencing depth across all samples was approximately 19×. After quality control and SNP calling, a total of 1,338,904 high-quality SNPs were identified and retained for subsequent analyses.

### 3.2. Three New Genetic Resources of A. cerana Identified in Sichuan

**Figure 2:**
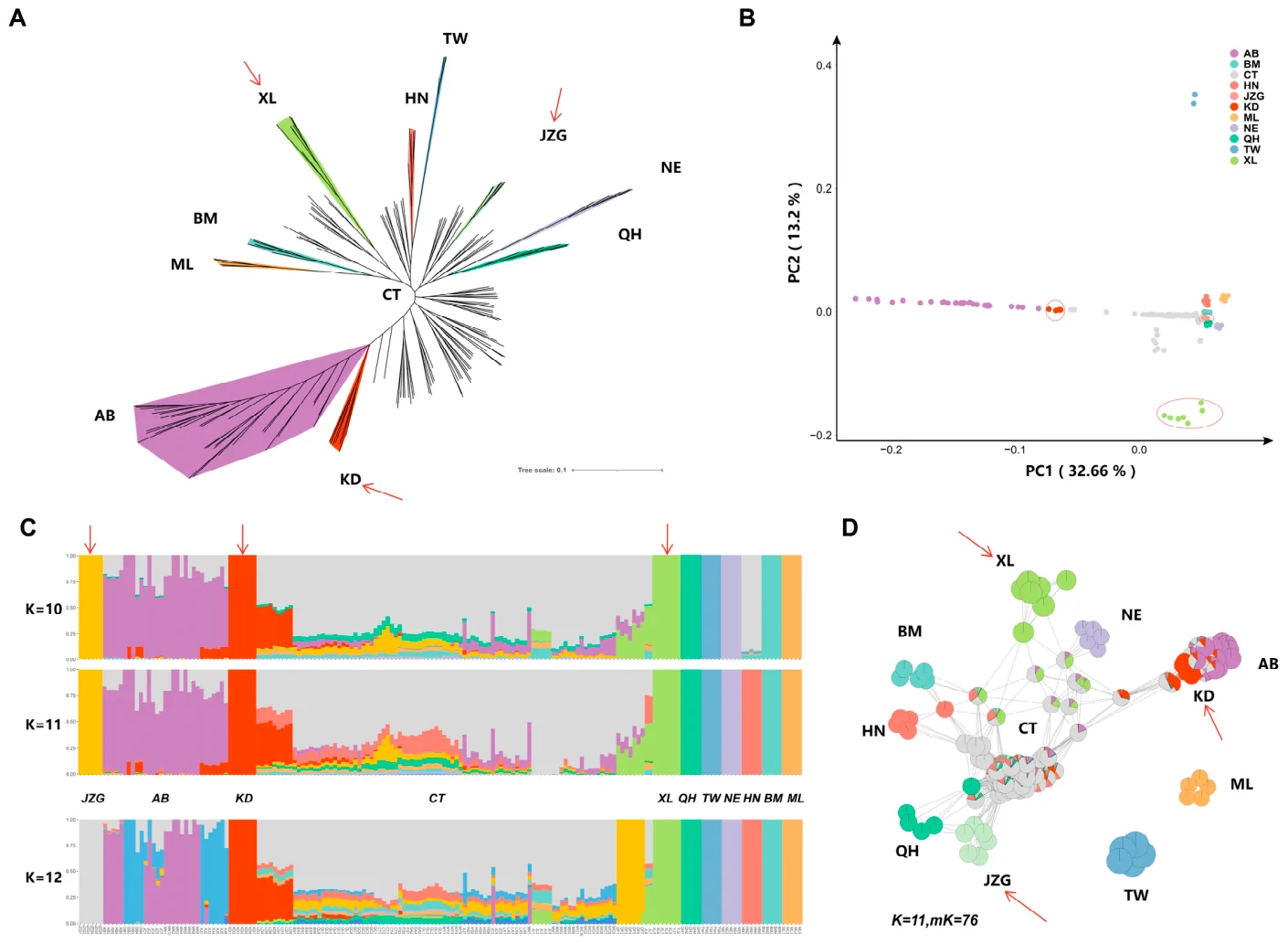
Population genetic structure of Sichuan *A. cerana* samples relative to the eight known subspecies resolved by genome-wide SNPs. Population abbreviations: XL, Xinlong; KD, Kangding; JZG, Jiuzhaigou; AB, Aba; BM, Bomi; CT, Central; HN, Hainan; QH, Qinghai; ML, Malaysia; NE, Northeast; TW, Taiwan. Red arrows or circles in the figure point to the three new genetic resources identified in this study. (**A**) Maximum likelihood phylogenetic tree constructed for the analyzed *A. cerana* samples; (**B**) Principal component analysis of the *A. cerana* samples; (**C**) ADMIXTURE analysis at K = 10, K = 11, and K = 12 for the analyzed *A. cerana* samples; (**D**) NetView network showing individual-level genetic relationships. In panels A, B, and D, colors represent different population groups; in panel C, different colors represent ancestry components inferred by ADMIXTURE.

To place the Sichuan *A. cerana* samples within the existing genetic reference framework for continental *A. cerana*, genomic data from Sichuan were analyzed jointly with publicly available genomic datasets. The resulting phylogenetic tree showed that individuals from each known subspecies (including Aba, Bomi, Hainan, Taiwan, Northeast, and Qinghai) clustered together and formed a distinct clade whose relationships trace back to the Central population (Figure 2A). This pattern is consistent with previous reports [12,13]. It is important to note that the phylogeny is an unrooted tree (Figure 2A) and the primary aim of this study is not to reconstruct the comprehensive structure of all Asian honey bees, but to determine whether any geographic populations in Sichuan Province represent distinct genetic resources. Within this framework (Figure 2A), most Sichuan samples clustered with known subspecies. In contrast, samples from Xinlong, Jiuzhaigou, and Kangding formed three clusters that were distinct from all known subspecies (Figure 2A), suggesting that they may represent previously unrecognized genetic resources.

Principal component analysis (Figure 2B) further showed that the Xinlong population was clearly separated from all other *A. cerana* samples along the second principal component (PC2). The Kangding and Jiuzhaigou populations occupied adjacent positions between the Central, Aba, and other reference populations, yet each retained a relatively tight local cluster (Figure 2B). The individual-level genetic relationship network generated by NetView showed that the Xinlong and Jiuzhaigou populations formed distinct peripheral clusters, indicating clear network separation (Figure 2D). Although the Kangding population lay near the Aba cluster, consistent with a relatively close genetic relationship to Aba, it maintained local clustering and was not fully integrated into the network core of either the Aba or Central populations (Figure 2D). Finally, ADMIXTURE analysis resolved all eight known *A. cerana* subspecies at K = 11. At this same K value, the Kangding, Jiuzhaigou, and Xinlong populations each displayed a genetic component that was distinct from all eight known subspecies, including the ancestral Central subspecies and the Sichuan-region Aba subspecies (Figure 2C).

Taken together, the phylogenetic relationships, PCA, NetView networks, and ADMIXTURE results consistently indicate that samples from Xinlong, Jiuzhaigou, and Kangding of Sichuan Province exhibit distinct population structures and should represent three new genetic resources of *A. cerana*.

### 3.3. Nucleotide Diversity and Linkage Disequilibrium Decay

To compare levels of within-population genetic variation, genome-wide average nucleotide diversity (π) was calculated using a sliding-window approach for four populations: the Jiulong population (collected in Sichuan Province and clustered with the Central population) and the three newly identified Xinlong, Jiuzhaigou, and Kangding populations. The Jiulong population exhibited the highest nucleotide diversity (π = 0.00429197), whereas the Xinlong, Jiuzhaigou, and Kangding populations showed lower values (π = 0.00332350, 0.00376833, and 0.00397624, respectively). These results are consistent with previous findings that the Central group harbors higher genetic diversity than peripheral groups [12].

**Figure 3:**
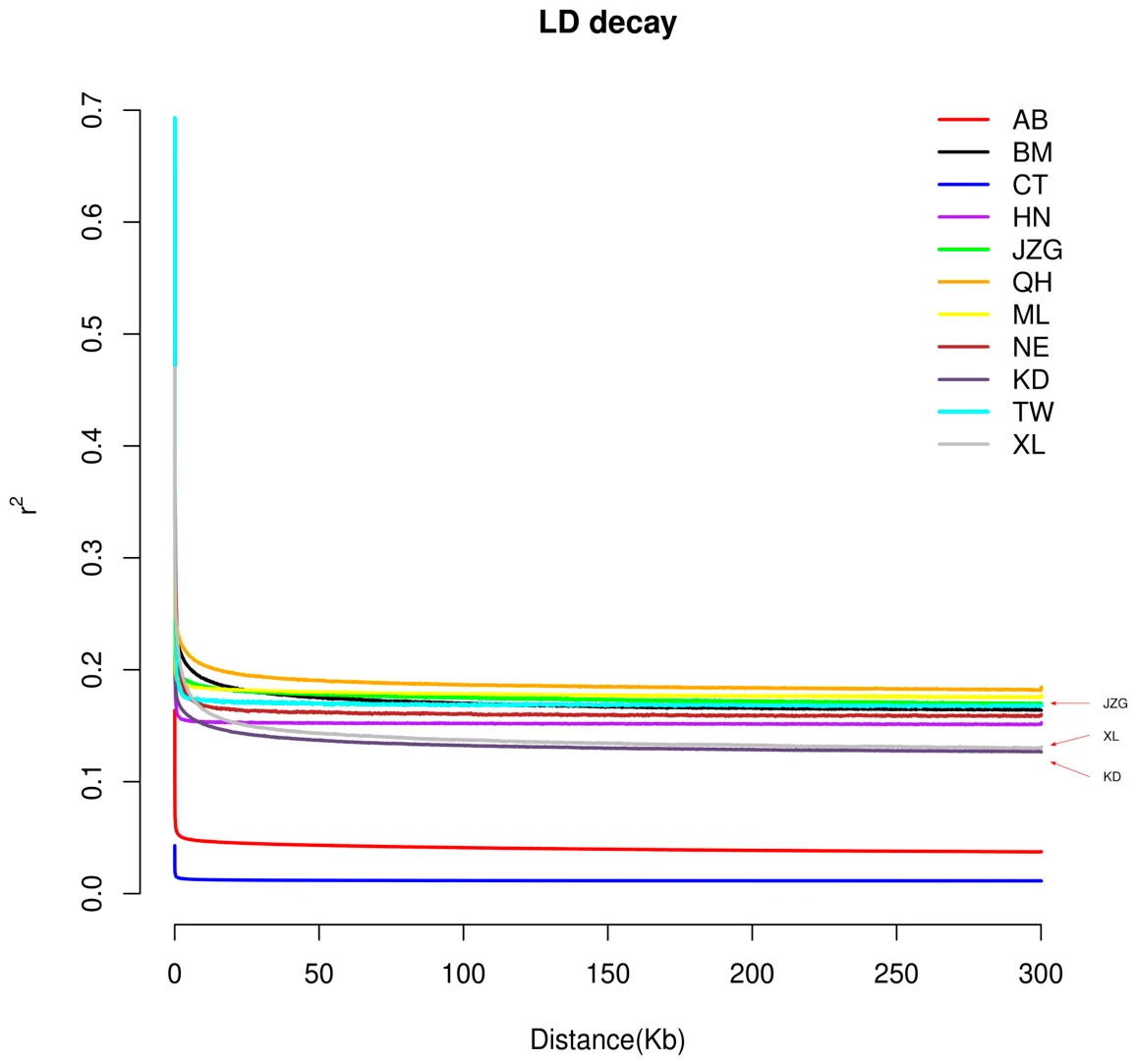
Linkage disequilibrium decay of Sichuan *A. cerana* populations and eight known subspecies. The *x*-axis represents the physical distance between SNP pairs, and the *y*-axis represents the squared correlation coefficient (*r*^2^). Population abbreviations: XL, Xinlong; KD, Kangding; JZG, Jiuzhaigou; AB, Aba; BM, Bomi; CT, Central; HN, Hainan; QH, Qinghai; ML, Malaysia; NE, Northeast; TW, Taiwan.

Linkage disequilibrium (LD) decay analysis likewise showed that the Central population had the fastest LD decay, whereas the Xinlong, Jiuzhaigou, and Kangding populations exhibited much slower decay, patterns similar to those of other peripheral groups except Aba (Figure 3). The slower LD decay further supports the classification of the three newly identified genetic resources as peripheral populations of *A. cerana*.

### 3.4. Genetic Differentiation Between the Three New Genetic Resources and Known Subspecies

**Figure 4:**
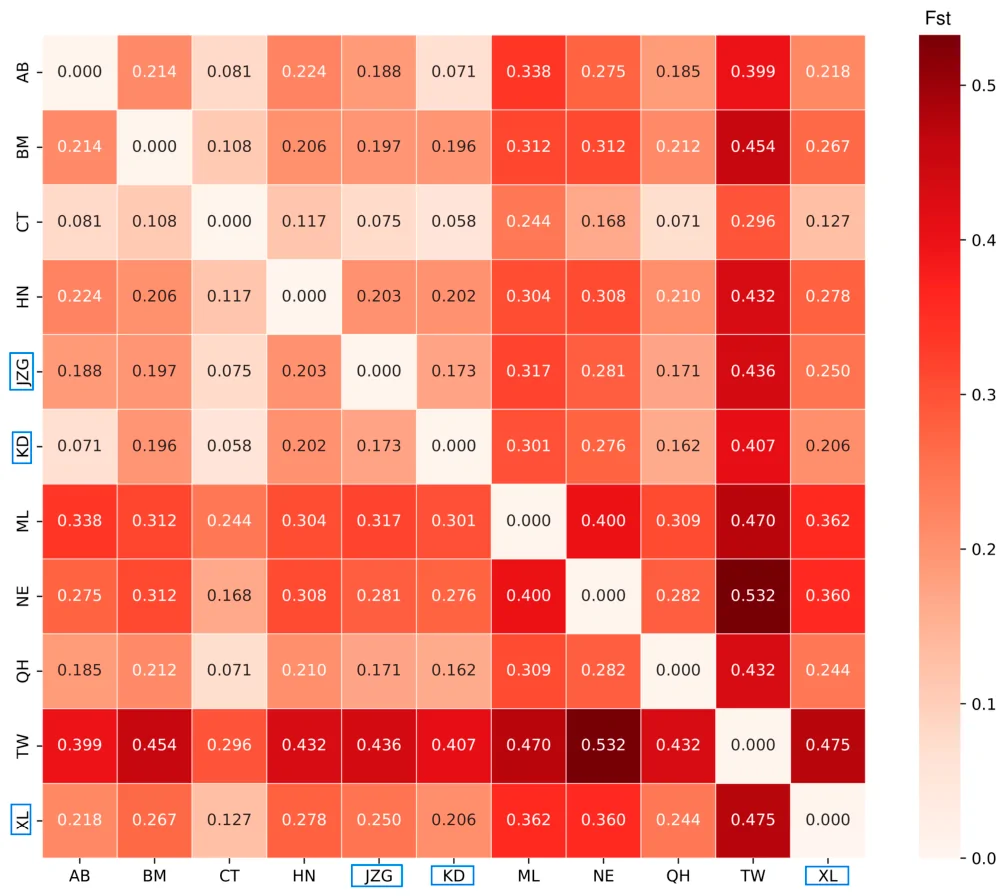
Pairwise genetic differentiation among three Sichuan *A. cerana* populations and eight known subspecies based on genome-wide SNP data. The heatmap shows pairwise *F*_ST_ values, with darker red colors indicating higher levels of genetic differentiation. Population abbreviations: XL, Xinlong; KD, Kangding; JZG, Jiuzhaigou; AB, Aba; BM, Bomi; CT, Central; HN, Hainan; QH, Qinghai; ML, Malay; NE, Northeast; TW, Taiwan. Blue boxes highlight the three Sichuan populations identified in this study (XL, KD, and JZG).

Pairwise *F*_ST_ analysis revealed varying degrees of genetic differentiation between the three newly identified Sichuan populations and the known subspecies (Figure 4). The *F*_ST_ between Xinlong and the Central population was 0.127 (moderate differentiation), whereas values between Xinlong and the other known subspecies ranged from 0.218 to 0.475 (high to extremely high differentiation). The Jiuzhaigou population showed relatively low differentiation from the Central population (*F*_ST_ = 0.075; still moderate) but high to extremely high differentiation from the remaining subspecies (*F*_ST_ = 0.171–0.436). The Kangding population exhibited the lowest differentiation from the Central population (*F*_ST_ = 0.058; moderate) and, apart from moderate differentiation from Aba, showed high to extremely high differentiation from the other subspecies (*F*_ST_ = 0.185–0.339). Clear differentiation was also observed among the three new populations themselves: pairwise *F*_ST_ values for Kangding–Jiuzhaigou, Kangding–Xinlong, and Jiuzhaigou–Xinlong were 0.173, 0.206, and 0.250, respectively, indicating high genetic differentiation. Collectively, the three new *A. cerana* genetic resources display moderate to high levels of genetic differentiation from known subspecies, further supporting their genetic distinctiveness.

### 3.5. Historical Changes in Effective Population Size

**Figure 5:**
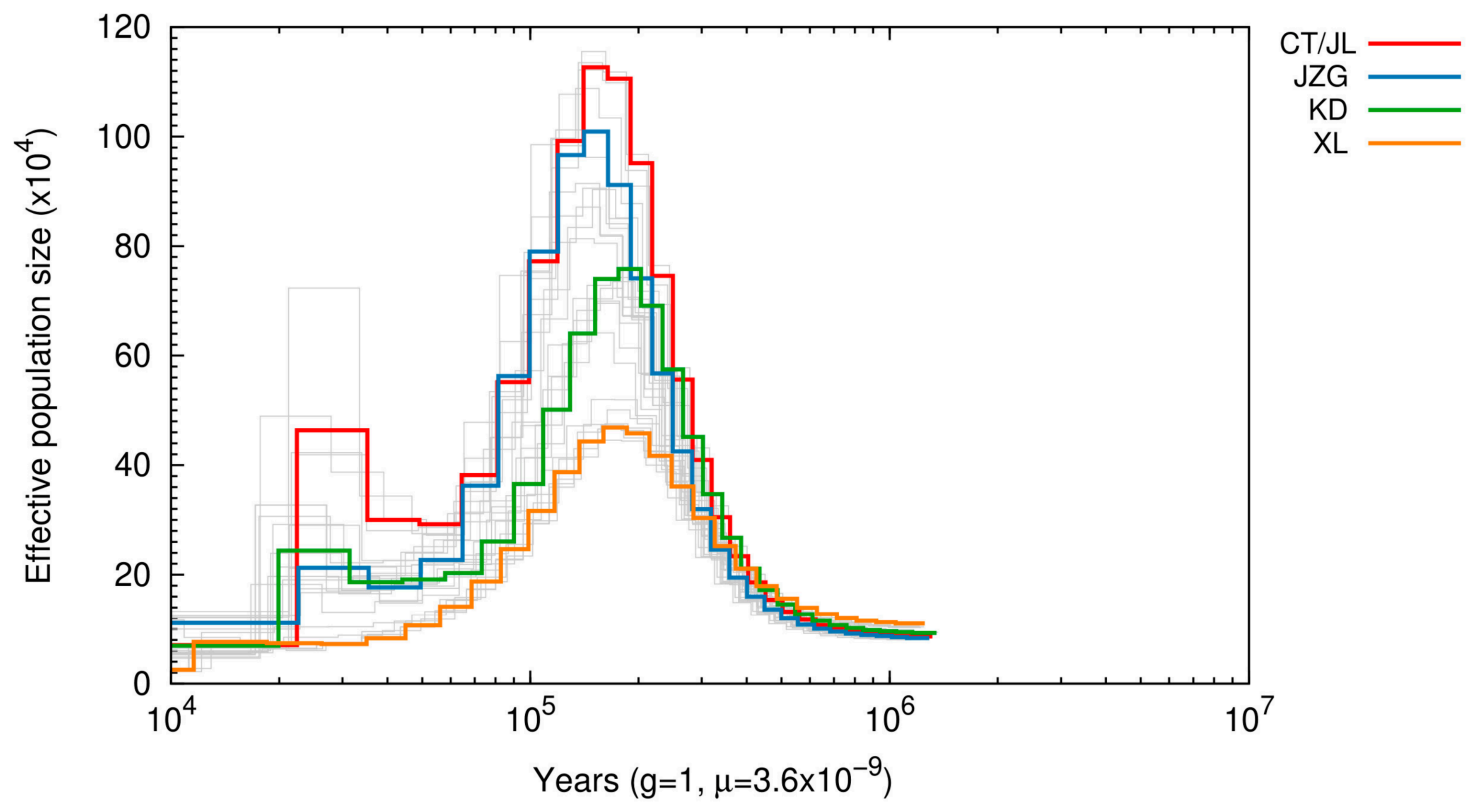
Historical changes in effective population size inferred using the PSMC model. The analysis was performed for CT/JL, JZG, KD, and XL, with JL denoting the Jiulong population representing the Central population. Gray lines indicate individual-level PSMC trajectories, and colored lines indicate population-level trajectories. Time was scaled using a generation time of 1 year and a mutation rate of 3.6 × 10^−9^ per site per generation. Population abbreviations: XL, Xinlong; KD, Kangding; JZG, Jiuzhaigou; CT, Central.

PSMC analysis revealed that the Jiulong population (sampled in Sichuan and representing the Central population), together with the Jiuzhaigou, Kangding, and Xinlong populations, shared broadly similar trajectories of historical effective population size (*N*_e_), with peaks occurring approximately 1.3–1.8 × 10^5^ years ago, followed by a subsequent decline. Nevertheless, clear differences in absolute *N*_e_ levels were evident among the four populations (Figure 5). The Jiulong population maintained the highest *N*_e_ throughout most of the historical period and also exhibited the highest peak value. The *N*_e_ of the Kangding population was markedly lower than that of Jiulong during most intervals, whereas the largest disparity relative to Jiulong was observed in the Xinlong population, which consistently showed the lowest *N*_e_ and a peak far below that of Jiulong. The Jiuzhaigou population tracked Jiulong closely during most periods but exhibited a markedly lower *N*_e_ from approximately 5 × 10^4^ to 2.3 × 10^4^ years ago, corresponding to late Marine Isotope Stage (MIS) 3 through early MIS 2.

### 3.6. Genes Involved in the Adaptive Evolution in the Three Distinct Populations

**Figure 6:**
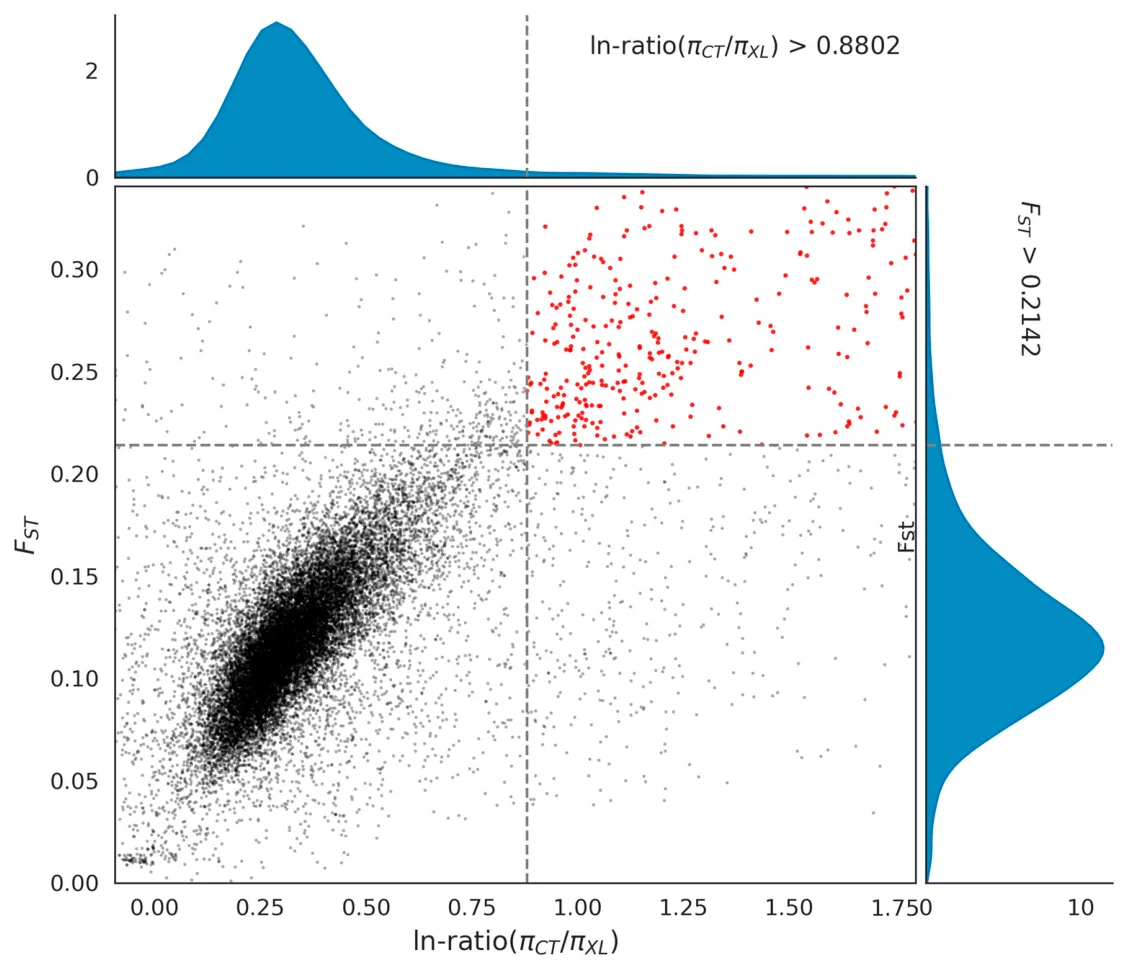
Distribution of candidate genomic regions detected by selective sweep analysis based on *F*_ST_ and the nucleotide diversity ratio between CT and XL. Gray points represent genomic windows, and red points indicate candidate regions that passed the top 5% thresholds for both *F*_ST_ and ln(π_CT_/π_target_). Dashed lines indicate the corresponding thresholds, and marginal density plots show the empirical distributions of each statistic. The blue shaded areas represent the marginal density distributions of the two statistics.

To identify candidate genomic regions that may be involved in the adaptive evolution of these three distinct Sichuan populations, the Central population was used as background to perform selective sweep analysis. Joint scans based on *F*_ST_ and ln(π_CT_/π_target_) were performed separately for the Jiuzhaigou, Kangding, and Xinlong populations (Figure 6 and Figures S1 and S2). These results were integrated with CLR scans from SweeD, and only overlapping regions supported by both the inter-population joint scan and the within-population CLR analysis were retained as high-confidence candidate regions. Genes located within these regions were believed to be involved in the adaptive evolution of these populations.

**Figure 7:**
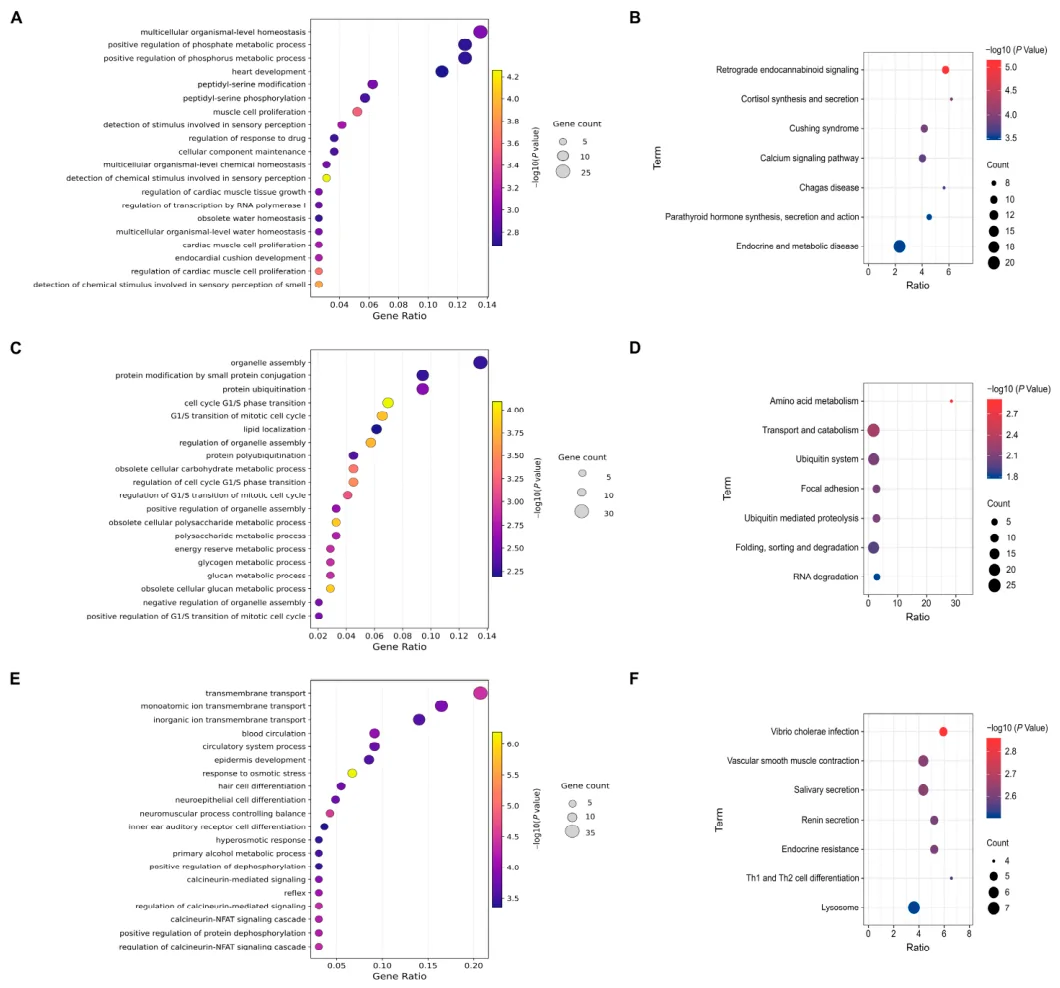
GO and KEGG enrichment analyses of candidate genes under selection in the three Sichuan *A. cerana* populations. (**A**) GO enrichment analysis of the Xinlong population (XL); (**B**) KEGG enrichment analysis of the Xinlong population (XL); (**C**) GO enrichment analysis of the Jiuzhaigou population (JZG); (**D**) KEGG enrichment analysis of the Jiuzhaigou population (JZG); (**E**) GO enrichment analysis of the Kangding population (KD); (**F**) KEGG enrichment analysis of the Kangding population (KD). The *x*-axis represents the gene ratio or enrichment ratio, and the *y*-axis represents enriched GO terms or KEGG pathways. Bubble size indicates the number of candidate genes assigned to each term or pathway, and color indicates the −log_10_-transformed *p* value.

A total of 202 candidate genes were identified in the Xinlong population (Table S3). GO and KEGG functional enrichment analyses were performed on these genes (*p* < 0.05; Tables S4 and S7). In the biological-process (BP) category, GO terms were enriched for “detection of chemical stimulus involved in sensory perception” and “muscle cell proliferation” (Figure 7A; Table S4). KEGG enrichment analysis showed significant enrichment in pathways such as “retrograde endocannabinoid signaling,” “calcium signaling pathway,” and “taste transduction” (Figure 7B; Table S7). The Xinlong sampling site lies at a high elevation of 3250 m (Table S1) and is characterized by pronounced vertical vegetation differentiation and spatially patchy nectar-plant resources. Chemical perception is fundamental to floral-resource recognition, foraging decisions, and navigation in honey bees [43,44,45]. Flight muscles are the most developed musculature in honey bees and power wing vibration and flight [46]. The KEGG pathways “retrograde endocannabinoid signaling” and “calcium signaling pathway” are involved in Ca^2+^-mediated neural signal regulation and cellular signal transduction, both of which may contribute to neuromuscular control during flight. We therefore infer that selection on these genes is associated with adaptation to an environment in which nectar resources are relatively sparse and bees must undertake prolonged high-elevation flights in search of floral resources.

A total of 256 candidate genes were identified in the Jiuzhaigou population (Table S3). GO enrichment in the BP category highlighted terms including “cell cycle G1/S phase transition,” “regulation of organelle assembly,” and “cellular carbohydrate metabolic process” (Figure 7C; Table S5). KEGG enrichment analysis indicated enrichment primarily in “Amino acid metabolism” and “Ubiquitin system” (Figure 7D; Table S8). The Jiuzhaigou sampling site lies in an alpine region. Bee colonies in such habitats experience a short spring reproductive window and must expand rapidly within a limited period to exploit the brief honey-flow season and improve overwintering success [37,47]. Genes involved in the G1/S phase transition of the cell cycle are central to reproduction, growth, and development [48]; their enrichment under selection is therefore consistent with selection for accelerated nurse- and worker-bee production. We infer that these candidate genes contribute to adaptation to a relatively short and concentrated honey-flow period.

A total of 175 candidate genes were identified under selection in the Kangding population (Table S3). GO enrichment in the BP category recovered terms including “response to osmotic stress,” “neuromuscular process controlling balance,” “transmembrane transport,” and “regulation of calcineurin-NFAT signaling cascade” (Figure 7E; Table S6). KEGG enrichment analysis highlighted pathways such as “Lysosome,” “Salivary secretion,” “Vascular smooth muscle contraction,” “Renin secretion,” “Th1 and Th2 cell differentiation,” “Endocrine resistance,” and “*Vibrio cholerae* infection” (Figure 7F; Table S9). Kangding experiences a plateau-mountain climate with large elevational gradients and spatially patchy nectar resources. Among the enriched GO terms, “response to osmotic stress” and “transmembrane transport” relate to the maintenance of cellular osmotic balance; osmotic pressure is intimately linked to water–solute regulation in cells and body fluids [49], which itself responds to external temperature variation [50]. Honey bees also exhibit higher water turnover than most terrestrial insects, partly because of metabolic water production during flight [51]. Taken together, selection on these genes is likely associated with adaptation to environments that demand longer foraging flights and that experience large diurnal temperature fluctuations.

## 4. Discussion

Sichuan Province is characterized by complex topography and diverse climatic conditions that create a mosaic of habitats for organisms in this region, including the Asian honey bee. Such environmental heterogeneity may have promoted local differentiation and fostered distinct *A. cerana* genetic resources that would be missed without dense geographic sampling. In this study, we collected *A. cerana* from 22 locations across Sichuan Province and generated whole-genome resequencing data for these samples. By analyzing this dataset together with representative genomes from the eight well-established *A. cerana* subspecies, we asked whether previously unrecognized genetic resources exist in Sichuan.

Thousands of sequenced *A. cerana* genomes are now available in public databases, yet they represent only the eight known subspecies [12,13,14,37,39]. Incorporating every published genome into each downstream analysis would impose substantial computational and storage costs and is unnecessary for the question addressed here. Accordingly, when comparing Sichuan samples with known subspecies, we randomly selected five individuals from each subspecies to serve as a reference panel. In the resulting phylogeny, individuals from each known subspecies clustered together and formed a distinct clade whose relationships trace back to the Central population (Figure 2A). This broad pattern is consistent with previous reports, although relationships among subspecies showed some inconsistency with trees inferred from much larger datasets [12,13]. These results indicate that the reduced reference panel is sufficient to distinguish genetically distinct *A. cerana* lineages, even if the overall topology has somewhat lower resolution than analyses based on the full public dataset. To further evaluate the impact of the limited sample size from the Central population on the identification of new genetic resources, we conducted a repeat analysis using 30 individuals from the Central population (Table S12). The results demonstrated that the Xinlong, Jiuzhaigou, and Kangding populations continued to be distinct genetic groups (Figure S3), thereby reinforcing the robustness of our findings.

Importantly, the inference of novel lineages does not rest on phylogeny alone. Principal component analysis, ADMIXTURE, network analyses, and genetic differentiation consistently identified samples from Xinlong, Jiuzhaigou, and Kangding as distinct from the eight known subspecies (Figure 2 and Figure 4). This concordance across independent methods substantially strengthens the case that the three Sichuan groups represent previously unrecognized genetic resources rather than artifacts of a particular analysis.

To test whether the five-individual sampling scheme introduced systematic bias into pairwise differentiation estimates, we compared all 28 pairwise *F*_ST_ values among the eight reference populations that were shared between this study and a previous analysis based on a larger sample [13]. The two datasets were highly consistent (Pearson *r* = 0.970; MAE = 0.0234; RMSE = 0.0302; mean signed difference = −0.0027), with no obvious overall upward or downward bias (Tables S10 and S11). Thus, although using only five individuals per reference population may reduce resolution to some extent, it does not appear to distort the relative genetic distances among known subspecies. Taken together, the multi-method concordance and the quantitative agreement of *F*_ST_ estimates support the conclusion that the Xinlong, Jiuzhaigou, and Kangding populations represent three reliable new genetic resources of *A. cerana*. However, to better investigate the taxonomic status of these new genetic resources—for example, whether they represent new subspecies—as well as the molecular mechanisms of their environmental adaptation, more honey bee individuals should be collected in the future for sequencing and phenotypic measurements.

### 4.1. Geographic Barriers and Ecological Differences May Jointly Contribute to Genetic Differentiation

Pairwise *F*_ST_ analysis among populations revealed the degree of genetic differentiation between the three newly identified populations and the known subspecies. Except for moderate genetic differentiation between the three new populations and the Central population, as well as between the Kangding and Aba populations, the three new populations showed high to extremely high differentiation from the other peripheral subspecies (Figure 4). However, because morphological data are currently lacking, whether these three new genetic resources represent new subspecies still requires further validation.

Restricted gene flow can lead to genetic differentiation. For social insects such as honey bees, effective gene flow among populations depends primarily on mating between queens and drones and on the dispersal and successful establishment of new colonies, rather than on routine foraging flights by worker bees [52]. In *A. cerana*, drones congregate at specific drone congregation areas, where queens mate; thus, genetic exchange is constrained by the movement ranges and encounter opportunities of reproductive individuals. High-elevation mountains, ridges, plateau surfaces, and other geographic barriers may reduce such encounters between populations, even though individual bees are capable of flight. In addition, the successful establishment of newly dispersed colonies is influenced by the availability of suitable nesting sites and food resources [53]. Therefore, geographic barriers may reduce mating opportunities and restrict cross-regional dispersal and successful establishment of new colonies, thereby impeding effective gene flow among local populations.

In our study, the Xinlong sampling site lies in the high-mountain canyon region of the middle Yalong River in Xinlong County, Ganzi Prefecture, adjacent to the Yalong River valley. The valley runs roughly north–south and is flanked to the east and west by continuous high-elevation mountains and snow-line ridges; the valley itself is narrow, and the terrain is highly rugged. The Jiuzhaigou site occupies a high-mountain canyon and montane-forest environment in the Minshan Mountains, where ridges, valley slopes, and branch valleys are interspersed. The Kangding site is situated in a high-mountain canyon and mountain-slope setting on the eastern side of the Dadu River. To the west lie the north–south-oriented Dadu River valley and its road corridor, whereas the eastern, southeastern, and northeastern sides are dominated by rugged high mountains with interspersed ridges, valley slopes, and branch valleys. All three populations therefore occupy areas of marked geographic isolation. A previous population genomic study of *A. cerana* from Jiuzhaigou classified the local population as a relatively isolated mountain population and reported relatively high genetic differentiation from geographically nearby mountain populations (*F*_ST_ = 0.16) [14], further supporting the role of topographic barriers in restricting gene flow.

In addition to the effects of geographic barriers on gene flow, we also considered the possibility that ecological differences among regions may influence population genetic differentiation. A previous study on genetic differentiation in *A. cerana* found clear genetic differentiation between two populations even in the absence of obvious geographic isolation, and the authors ultimately suggested that local ecological differences impeded gene flow [37]. The three populations identified in our study are characterized by marked geographic barriers and also differ substantially in topography and landforms, climate, and nectar plant resources. Such ecological differences may have promoted long-term independent adaptation among populations, leading them toward different differentiation trajectories, although this inference requires further validation in future studies.

### 4.2. Environmental Adaptation of the Three Newly Identified Genetic Resources

In each of the three populations, hundreds of genes exhibit signs of positive selection (Table S3), likely reflecting their adaptation to patchy nectar resources, a short spring reproductive window, and significant diurnal temperature variations within their habitats. Furthermore, all three populations display signals associated with flight and neuromuscular function; however, the molecular pathways involved differ among them: the XL population emphasizes energy metabolism and olfaction, while the JZG population focuses on cell cycle and proteostasis, and the KD population is associated with calcineurin and osmotic responses (Figure 7; Tables S4–S9). This indicates a convergent adaptation to challenging flight environments through distinct molecular targets in these three new genetic resources. Ultimately, the genes and genotypes identified within these populations could be harnessed to enhance related traits in other *A. cerana* subspecies.

### 4.3. Conservation Implications

*A. cerana* is facing an unprecedented decline in population diversity, which directly impairs crop pollination services in Asia and further exacerbates food shortage risks for humans [54,55]. Sichuan has complex terrain and diverse ecological types and has long preserved relatively rich local *A. cerana* resources. At the same time, Sichuan is also a region where *A. cerana* is actively utilized. In response to this situation, relevant authorities have established a conservation–utilization system centered on known local resources, including the Aba National Conservation Farm for Chinese Honey Bees, provincial-level conservation areas such as Maerkang, and core breeding farms [56,57]. However, these systems are mainly based on existing knowledge of resources and traditional conservation targets, and their coverage of finer-scale genetic differentiation within the region remains limited.

Based on whole-genome data, this study identified three relatively differentiated local genetic populations in Sichuan, namely the Xinlong, Jiuzhaigou, and Kangding populations. This indicates that in Sichuan, a region characterized by strong ecological heterogeneity and marked geographic separation, there are still local genetic resources that have not yet been fully incorporated into conservation planning. These populations showed a certain degree of distinctiveness in population structure, genetic differentiation, and candidate adaptive signals. In future conservation efforts, whole-genome genetic information should be incorporated into resource surveys and conservation planning for *A. cerana* in Sichuan. Continuous genetic monitoring should be conducted for local genetic populations such as Xinlong, Jiuzhaigou, and Kangding, and uncontrolled mixing among different genetic units should be avoided as much as possible during colony introduction, propagation, and cross-regional transportation.

Therefore, conservation of *A. cerana* in Sichuan should not focus only on maintaining colony numbers, but should also emphasize the identification of local genetic units, the maintenance of genetic integrity, and the preservation of local adaptive potential. This study provides genomic evidence for the refined identification and region-specific conservation of *A. cerana* genetic resources in Sichuan. It also suggests that conservation of native honey bees in ecologically complex regions needs to move beyond “resource preservation” toward “identification and conservation of potential resources.”

## 5. Conclusions

Based on extensive sampling across Sichuan and whole-genome resequencing data, this study systematically analyzed the population genetic structure, genetic differentiation, evolutionary differences, and genetic basis of environmental adaptation in Sichuan *A. cerana*. The results showed that the Xinlong, Jiuzhaigou, and Kangding populations each formed distinct local genetic populations and exhibited varying degrees of genetic differentiation from known *A. cerana* subspecies. These findings indicate that previously under-recognized local genetic resources of *A. cerana* are still preserved in the complex terrain and heterogeneous environments of Sichuan. However, because morphological data and additional ecological evidence are currently lacking, whether these three populations have distinct subspecies status requires further validation.

This study provides whole-genome evidence for the identification of new local genetic resources of *A. cerana* and reveals the genetic basis underlying their adaptation to local environments. It also suggests that, in complex mountain and valley environments, conservation of native honey bee populations should not focus only on maintaining colony numbers, but should also emphasize the identification of local genetic units, the maintenance of genetic integrity, and the preservation of local adaptive potential. Future conservation efforts should strengthen continuous genetic monitoring of local populations such as Xinlong, Jiuzhaigou, and Kangding, and avoid uncontrolled mixing among different genetic units during colony introduction, propagation, and cross-regional transportation.

## Data Availability

The raw whole-genome resequencing data generated in this study have been deposited in the Genome Sequence Archive (GSA) under accession number CRA046892.

## Supplementary Materials

The following supporting information can be downloaded at: https://www.mdpi.com/article/10.3390/insects17101022/s1, Figure S1: Distribution of candidate genomic regions detected by selective sweep analysis based on FST and the nucleotide diversity ratio between CT and JZG; Figure S2: Distribution of candidate genomic regions detected by selective sweep analysis based on FST and the nucleotide diversity ratio between CT and KD; Figure S3: Maximum likelihood phylogenetic tree of A. cerana samples constructed using 30 individuals from the Central population; Table S1: Sampling information for newly sequenced *A. cerana* samples from Sichuan Province; Table S2: Publicly available whole-genome resequencing data used in this study; Table S3: Candidate genes under selection identified in the XL, JZG, and KD populations; Table S4: GO enrichment results of candidate genes under selection in XL; Table S5: GO enrichment results of candidate genes under selection in JZG; Table S6: GO enrichment results of candidate genes under selection in KD; Table S7: KEGG enrichment results of candidate genes under selection in XL; Table S8: KEGG enrichment results of candidate genes under selection in JZG; Table S9: KEGG enrichment results of candidate genes under selection in KD; Table S10: Comparison of pairwise FST estimates among eight reference populations between the present study and the previous study [13]; Table S11: Agreement metrics for the 28 pairwise FST estimates between the present study and the previous study [13]; Table S12: Sample information for 30 individuals from the Central population used in the repeat analysis.
