# Supplementary material for: Population Genomic Insights into Genetic Differentiation and Environmental Adaptation of the Asian Honey Bee (Apis cerana) in Sichuan, China
Source: Insects. 2026 Oct 3;17(10):1022. doi: 10.3390/insects17101022 (PMC13705124; doi:10.3390/insects17101022)
Supplement: Supplementary file 1 [file insects-17-01022-s001.zip › Supplementary_Figures_Xu_et_al.pdf]

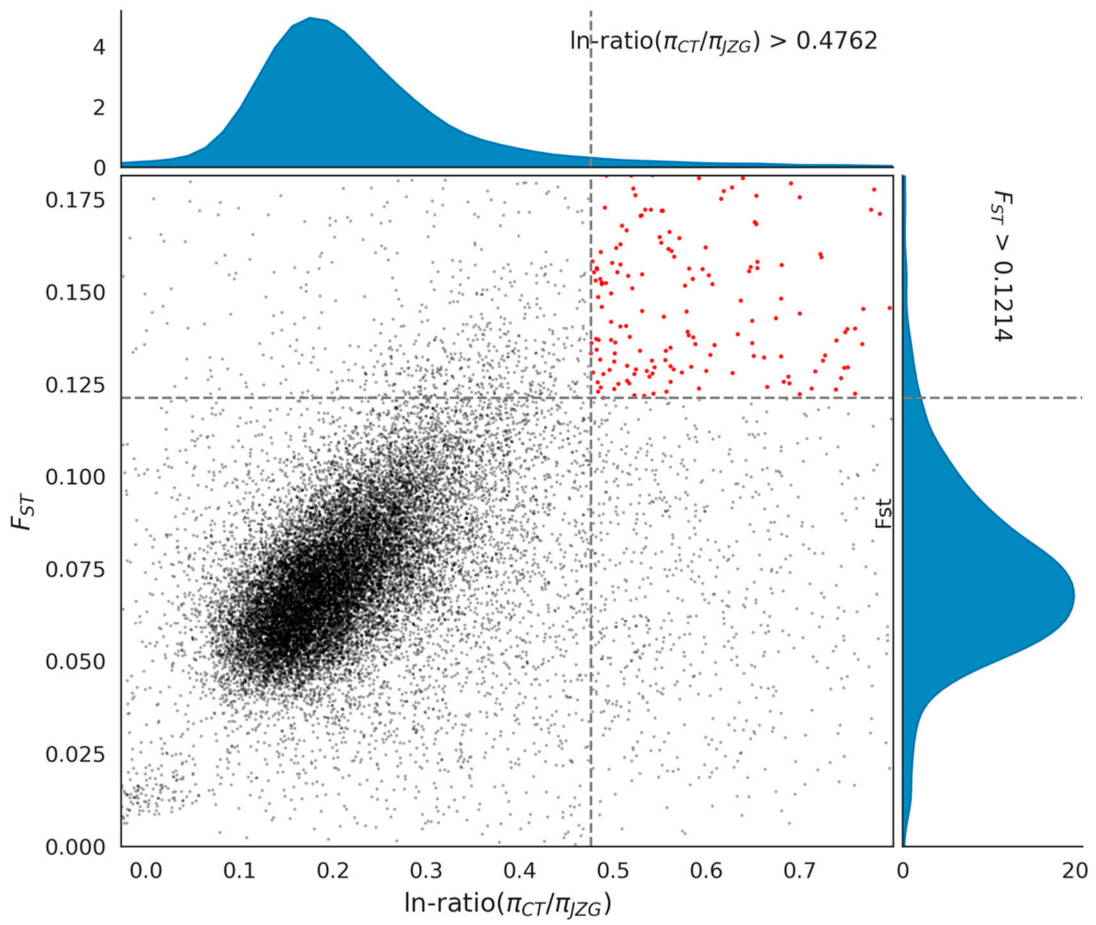

Figure S1. Distribution of candidate genomic regions detected by selective sweep analysis based on F_{ST} and the nucleotide diversity ratio between CT and JZG. Gray points represent genomic windows, and red points indicate candidate regions that passed the top 5% thresholds for both F_{ST} and $\ln(\pi_{\text{CT}}/\pi_{\text{target}})$. Dashed lines indicate the corresponding thresholds, and marginal density plots show the empirical distributions of each statistic. The blue shaded areas represent the marginal density distributions of the two statistics.

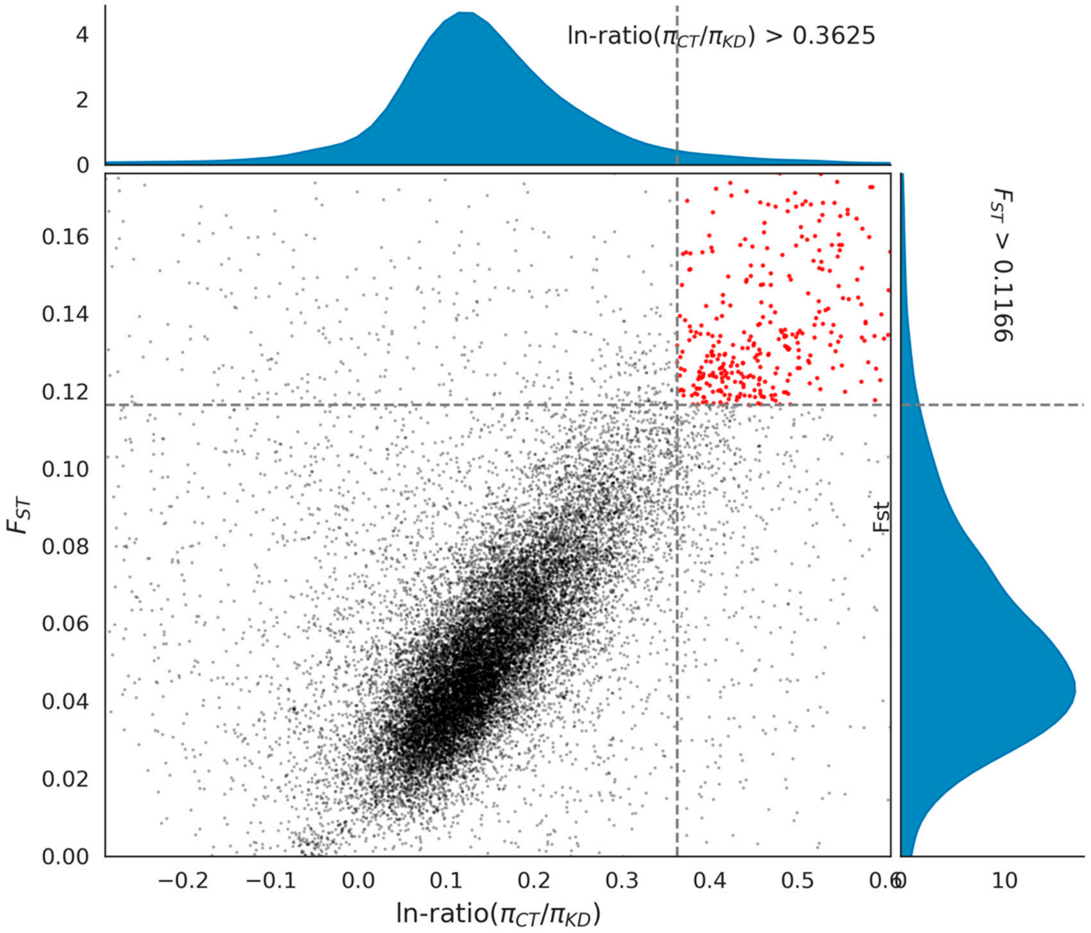

Figure S2. Distribution of candidate genomic regions detected by selective sweep analysis based on F_{ST} and the nucleotide diversity ratio between CT and KD. Gray points represent genomic windows, and red points indicate candidate regions that passed the top 5% thresholds for both F_{ST} and $\ln(\pi_{CT}/\pi_{target})$. Dashed lines indicate the corresponding thresholds, and marginal density plots show the empirical distributions of each statistic. The blue shaded areas represent the marginal density distributions of the two statistics.

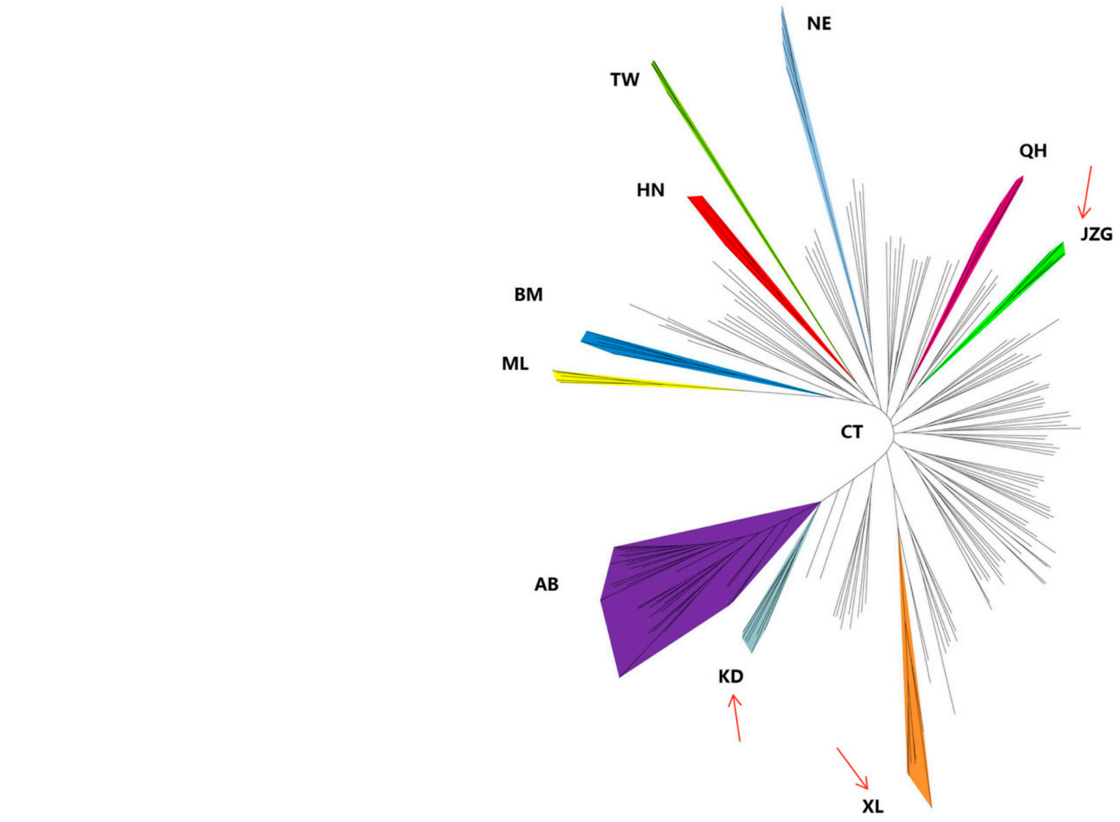

Figure S3. Maximum likelihood phylogenetic tree of A. cerana samples constructed using 30 individuals from the Central population. Population abbreviations: XL, Xinlong; KD, Kangding; JZG, Jiuzhaigou; AB, Aba; BM, Bomi; CT, Central; HN, Hainan; QH, Qinghai; ML, Malaysia; NE, Northeast; TW, Taiwan. Red arrows in the figure point to the Xinlong, Jiuzhaigou, and Kangding populations. Colors represent different population groups.
