# Supplementary material for: Population Genomic Insights into Genetic Differentiation and Environmental Adaptation of the Asian Honey Bee (Apis cerana) in Sichuan, China
Source: Insects. 2026 Oct 3;17(10):1022. doi: 10.3390/insects17101022 (PMC13705124; doi:10.3390/insects17101022)
Supplement: Supplementary file 1 [file insects-17-01022-s001.zip › Supplementary_Tables_S10-S11.pdf]

Table S10. Comparison of pairwise F_{ST} estimates among eight reference populations between the present study and the previous study [13]

Population 1	Population 2	Previous study [13] F_{ST}	Present study F_{ST}	Difference (ΔF_{ST})
**AB**	BM	0.23	0.214	−0.016
	CT	0.12	0.081	−0.039
	HN	0.24	0.224	−0.016
	ML	0.35	0.338	−0.012
	NE	0.31	0.275	−0.035
	QH	0.22	0.185	−0.035
	TW	0.39	0.399	+0.009
**BM**	CT	0.11	0.108	−0.002
	HN	0.22	0.206	−0.014
	ML	0.33	0.312	−0.018
	NE	0.31	0.312	+0.002
	QH	0.21	0.212	+0.002
	TW	0.39	0.454	+0.064
**CT**	HN	0.11	0.117	+0.007
	ML	0.22	0.244	+0.024
	NE	0.19	0.168	−0.022
	QH	0.09	0.071	−0.019
	TW	0.26	0.296	+0.036
**HN**	ML	0.34	0.304	−0.036
	NE	0.31	0.308	−0.002
	QH	0.21	0.210	0.000
	TW	0.37	0.432	+0.062
**ML**	NE	0.42	0.400	−0.020
	QH	0.35	0.309	−0.041
	TW	0.50	0.470	−0.030
**NE**	QH	0.29	0.282	−0.008
	TW	0.46	0.532	+0.072
**QH**	TW	0.42	0.432	+0.012

Note. $\Delta F_{ST} = F_{ST}$, present study − F_{ST}, previous study. Positive values indicate higher estimates in the present study, whereas negative values indicate lower estimates. Agreement metrics based on these 28 pairwise comparisons are summarized in Table S11.

Table S11. Agreement metrics for the 28 pairwise F_{ST} estimates between the present study and the previous study [13].

Metric	Statistical meaning	Calculation from the 28 pairwise comparisons	Value
Pearson correlation coefficient (r)	Linear correlation between the two sets of pairwise F_{ST} estimates	$\bar{x}$ = 0.284643; $\bar{y}$ = 0.281964; 0.347455 / √(0.323096 × 0.397231)	0.970
Mean absolute error (MAE)	Mean magnitude of the pairwise differences, irrespective of direction	$\Sigma\|d_i\|$ = 0.655; 0.655 / 28	0.0234
Root mean square error (RMSE)	Square root of the mean squared pairwise differences	Σd_i^2 = 0.025619; √(0.025619 / 28)	0.0302
Mean signed difference	Mean directional difference (present study − previous study)	Σd_i = −0.075; −0.075 / 28	−0.0027

Note. n = 28 pairwise comparisons. Let x_i denote the pairwise F_{ST} estimate from the previous study and y_i the corresponding estimate from the present study; $d_i = y_i - x_i$. All summations below are over i = 1, …, n. Pearson's $r = \Sigma[(x_i - \bar{x})(y_i - \bar{y})] / \sqrt{\{\Sigma(x_i - \bar{x})^2 \times \Sigma(y_i - \bar{y})^2\}}$; MAE = $(1/n)\Sigma|d_i|$; RMSE = $\sqrt{\{(1/n)\Sigma d_i^2\}}$; mean signed difference = $(1/n)\Sigma d_i$. The reported values were calculated directly from the 28 pairwise values shown in Table S10.
